# Attitudes and norms affecting scientists’ data reuse

**DOI:** 10.1371/journal.pone.0189288

**Published:** 2017-12-27

**Authors:** Renata Gonçalves Curty, Kevin Crowston, Alison Specht, Bruce W. Grant, Elizabeth D. Dalton

**Affiliations:** 1 Departamento de Ciência da Informação, Universidade Estadual de Londrina, Londrina, PR, Brazil; 2 School of Information Studies, Syracuse University, Syracuse, NY, United States of America; 3 Centre for the Synthesis and Analysis of Biodiversity, Foundation for the Research on Biodiversity, Aix-en-Provence, France; 4 School for Earth and Environmental Sciences, the University of Queensland, Lucia, Queensland, Australia; 5 Departments of Biology and Environmental Science, Widener University, Chester, PA, United States of America; 6 Department of Communication Studies, Middle Tennessee State University; Murfreesboro, TN, United States of America; Indiana University Bloomington, UNITED STATES

## Abstract

The value of sharing scientific research data is widely appreciated, but factors that hinder or prompt the reuse of data remain poorly understood. Using the Theory of Reasoned Action, we test the relationship between the beliefs and attitudes of scientists towards data reuse, and their self-reported data reuse behaviour. To do so, we used existing responses to selected questions from a worldwide survey of scientists developed and administered by the DataONE Usability and Assessment Working Group (thus practicing data reuse ourselves). Results show that the perceived efficacy and efficiency of data reuse are strong predictors of reuse behaviour, and that the perceived importance of data reuse corresponds to greater reuse. Expressed lack of trust in existing data and perceived norms against data reuse were not found to be major impediments for reuse contrary to our expectations. We found that reported use of models and remotely-sensed data was associated with greater reuse. The results suggest that data reuse would be encouraged and normalized by demonstration of its value. We offer some theoretical and practical suggestions that could help to legitimize investment and policies in favor of data sharing.

## Introduction

Research data are the backbone of scientific discovery and technological innovation, and are regarded as the prime currency of science [1, 2]; the ‘building blocks’ of research [3]. In the past, hard-won research data was only shared among a few trusted and known colleagues. Increasingly though, researchers are expected to be accountable for taxpayers’ money and make publicly funded research data available to the wider community, e.g., by depositing them in digital repositories. This accountability has become one of the key drivers in the open science agenda and has been redefining the social contract of science in many countries, but especially in the UK and the US [4].

Sharing data is seen as key to improving data integrity [5, 6] and for enhancing transparency and reproducibility of the scientific enterprise [7, 8]. Sharing data may also have personal benefits to researchers, such as increasing citation rates [5, 8, 9].

Several incentives to increase data sharing have been established in the past decade. Many funding agencies, journal publishers, academic institutions and research organizations across the globe have implemented mandates for research data sharing, often to comply with new governmental directives [10, 11]. Others have called for cultural change to accompany technology change [12]. These developments have produced substantial growth in data availability via digital repositories [13].

While making data openly available does have inherent benefits, most scientists sharing data with people outside their research team do so because they expect it to be reused [14]. Substantial attention has been paid to attitudes towards data sharing and data sharing behaviour among scientists (e.g., [11, 14–16]), but less attention has been given to the practice of scientific data reuse, here defined as research in which some or all of the data analyzed were collected by others besides the reuser or members of his/her research team. In other words, for this paper we constrain data reuse to the usage of a dataset by someone other than the originator to deploy new research [17]. Reuse of existing data is argued to be beneficial for several reasons. Reusing data can be more economical [18–20]; enable research based on novel combinations of data [21]; provide opportunities for co-authorship [8]; and generally enhance scientific progress [6, 11].

However, considerable time and a range of skills are required to reuse data. For example, researchers attempting to reuse data report:

- difficulty to discover available and relevant data,
- inability to discern dataset content and hence suitability for analysis (e.g. due to a lack of metadata); and
- inability to determine the quality of the data [22].

Aside from these challenges, a decision to reuse might be influenced by disciplinary traditions, data type, reuse purposes, and type of inquiry [23]. Not all data are easily reusable. Data, for example, cannot be regarded as "free-floating" commodities nor theory-free ‘matters of fact’ which can be de-coupled from the contexts and relationships through which they were produced [24, 25]. This, and the fact that some disciplines are more receptive to research based on second-hand data than others [46, 37] are likely impose additional barriers for scientists who plan to reuse data. Despite these challenges, many scientists feel that the time spent on data reuse is time well spent [26].

While the skills and benefits of data reuse have been the object of a few studies (e.g., [27]), less is known about the factors that promote or deter data reuse. Knowledge of such factors is important to understand how to encourage the practice. The contribution of this paper is to develop a theoretical model of the factors predicting data reuse and to test their importance with empirical data. Specifically, we test the relationship between scientists’ beliefs and attitudes towards data reuse and their self-reported data reuse behaviour using responses to selected questions from a worldwide survey developed and administered by the Data Observation Network for Earth (DataONE) Usability and Assessment Working Group [28].

This analysis is based on a published dataset. It is thus itself an example of the phenomenon of data reuse, as to test our hypotheses we re-purposed the data beyond the scope of the original descriptive study. The steps taken and challenges faced offer an additional contribution to understanding data reuse.

## Theory development

The survey from which we have obtained the data for this study was originally designed for descriptive analysis and so did not draw explicitly on a theoretical framework in its design. However, to analyze the data for factors predictive of data reuse, we propose the Theory of Reasoned Action (TRA) [29] as a suitable theoretical lens to conceptualize the effect of attitudes of respondents towards reuse.

**Fig 1:**
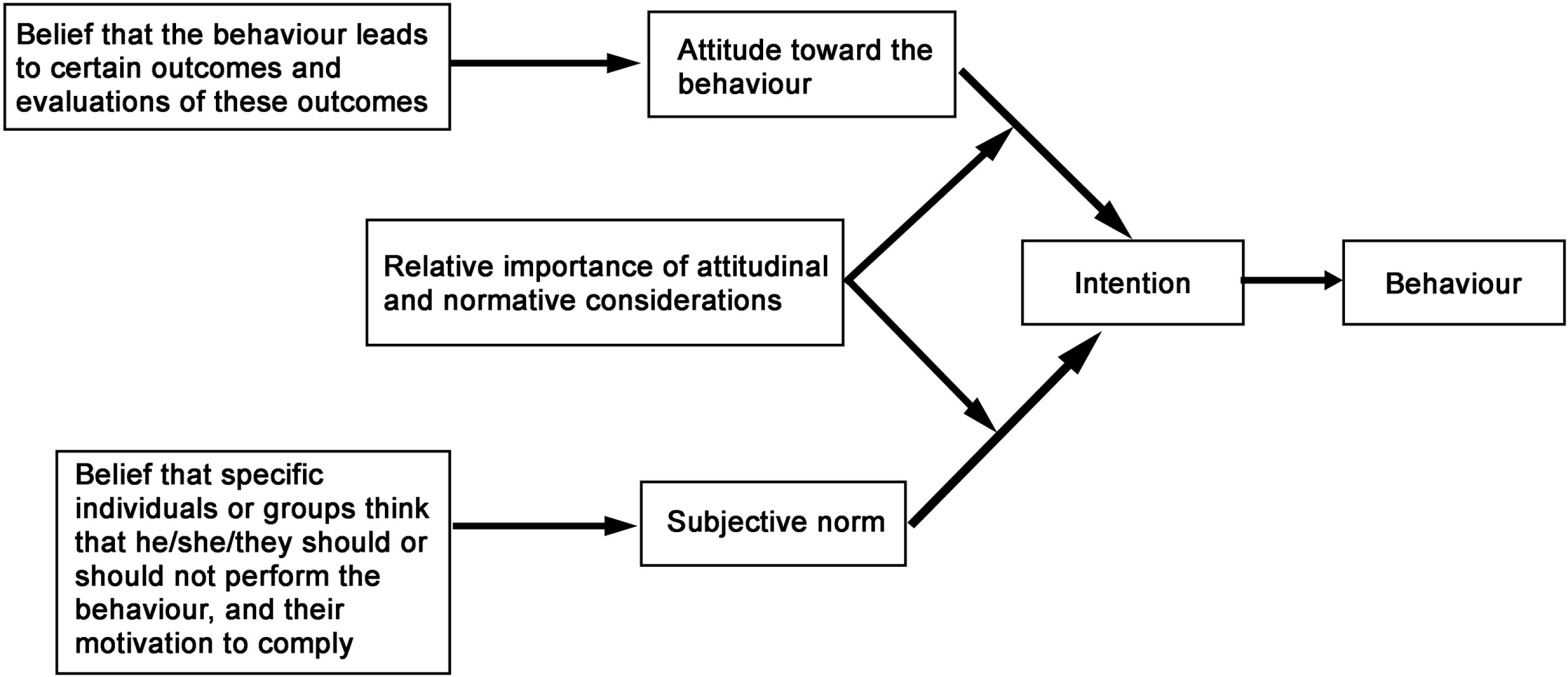
Factors determining a person’s behaviour using the Theory of Reasoned Action (adapted from [29]).

The TRA postulates that one’s intention to perform a given behaviour is the immediate predictor of one’s actual behaviour. This intention is driven in turn by two factors: (i) one’s attitudes toward the behaviour developed from an accrued understanding of the costs and benefits and value of the outcomes, and (ii) one’s perceptions of the immediate societal norms about the behaviour (Fig 1). The individual balances these two factors in making a decision of intent.

The TRA framework was chosen as several of the questions on the survey could be interpreted as measuring the key concepts of this theory: the respondents’ behaviours, reusing or not reusing data; their understandings of the potential risks and benefits of reusing data (attitudes); and their perceptions of the community’s feelings about the appropriateness of data reuse (subjective norms). We discuss these concepts as they relate to data reuse in turn.

### Attitudes towards data reuse

A person’s behaviour is a product of their attitudes. Attitudes in turn are shaped by individual experiences and represent combinations of enduring beliefs and feelings about socially constructed and significant objects, behaviour, groups or symbols [30]. According to the TRA, eliciting attitudes towards a given behaviour helps to predict the predisposition to perform such behaviour.

Attitudes can be assessed through positive and negative responses to a given behaviour, in this case positive or negative reactions to data reuse. Authors have described several advantages might scientists anticipate when they decide to reuse existing data as opposed to collecting new data (e.g., [9, 18, 31–34]). Data reuse has been advocated as a viable and parsimonious choice for researchers with limited time and resources [18–20]. Secondary data analysis has been suggested as a mechanism to lessen expenses related to data collection and to shorten the research process [35].

Additional positive aspects of data reuse that have been articulated include enabling studies to be extended beyond the temporal and spatial limitations of a single study [3, 36–40]; allowing trans-disciplinary applications [41, 42]; and enabling meta-analyses [5, 13, 14, 27, 43, 44].

Reusing data, however, poses risks that potentially trigger negative attitudes towards data reuse. First, data available for reuse may contain hidden errors that are not easily identifiable; raising the risk that effort will be wasted on flawed data [5, 20, 35]. A lack of control over data quality thus threatens a potential waste of time [22, 31]. Data reuse requires a great deal of trust in data producers, and heavy reliance on the methods and techniques they employed to obtain, organize and code the data.

Secondly, comprehensive metadata is needed to support the correct interpretation of the data. Scientists need to feel confident that they have enough information about the data to minimize the chances of making wrong assumptions and unintentionally misuse it [8, 14, 45]. A lack of such information—or just concern about such a lack—may lead scientists to refrain from reusing data they have not collected themselves or to have to spend a great deal of time to overcome the lack of metadata [8].

Finally, researchers may fear that they would have to adjust their own research design to be able to incorporate others’ data [46]. The context in which data were collected can greatly affect their suitability for reuse. The environment in which the data were acquired, the duration and frequency of collection, and the specific questions that were addressed, can all affect the nature and quality of the data for reuse. This mismatch can require additional time to align data sets for combination and reuse, countering the benefits of reuse.

### Subjective norms about data reuse

Subjective norms can be broadly defined as the social pressure one perceives and is influenced by to engage or not to engage in a behaviour. These represent the normative beliefs and perceptions individuals hold regarding the expectations of other people they regard highly [29]. These norms are labeled ‘subjective’ because they are not necessarily formally imposed upon individuals, but rather norms perceived by them. Peer pressure can outweigh individual willingness and motivations and hence guide behaviour. Data sharing behaviour has been found to be positively correlated to scientists’ perception about how receptive close colleagues and peers are to the practice [47].

As people are generally gregarious, they tend to form sub-cultures of similar-thinking people, and, at a smaller scale, cliques, consisting of groups of people who have similar sets of values and norms, and therefore similar attitudes and behavioural responses to stimuli [29, 48]. Scientists recognize themselves as a part of a scholarly ecosystem that functions based on ethos or norms [49] in favor of the advancement of science and the public good as a whole.

Scientists are also influenced by disciplinary traditions and group norms, which can be deliberately enforced, prompted or activated as subtle cues through observation of how others important to them behave. Peer influence and disciplinary traditions are sources of subjective norms, hereafter also referred to as perceived norms, that exert social pressure on scientists’ data sharing and reuse behaviour [15]. The perceived receptiveness, encouragement and recognition from peers and disciplinary communities to data reuse are key for scientists when they consider reuse as an option for their research [23, 42, 50, 51].

Researchers have found that some fields are more receptive than others to sharing due to the disciplinary variation of artifacts and the practices of scholarship [23]. For example, within the sciences, environmental and earth scientists are said to be conspicuous in their lack of data sharing ‘readiness’ [51]. In this paper, we consider the subjective norms derived from disciplinary and social influences as well as data types as potential predictors of scientists’ data reuse behaviour.

In summary, based on the TRA, we state the following proposition that conceptually guide this paper:

Scientists’ attitudes (e.g. perceived benefits and risks) and subjective norms towards data reuse influence their own data reuse behaviour.

A common theme in the literature about scientific data reuse is that scientists must be knowledgeable about data to be able to successfully reuse shared data. Awareness and knowledge of how to handle metadata and data documentation are important because they minimize frictions associated with data discoverability and understandability [43, 44]. To account for this factor, we consider how experience with data management affected scientists’ data reuse and how the effects of various attitudes and subjective norms might differ between more and less experienced scientists.

## Methods

The study from which we derived our data was a cross-sectional survey. As we were relying on data previously collected, the major effort in the analysis was to connect the available data to the concepts of interest discussed above and to use them to develop and test the specific hypotheses that addressed our research proposition.

### Dataset description and provenance

The dataset used in this study was collected by the DataONE Usability and Assessment Working Group to capture scientists’ attitudes towards data sharing, the data sharing practices in which they engage, satisfaction with different stages of the research lifecycle and perceptions of organizational support for research processes. The survey also included an optional section containing four questions about data reuse, the subject of this paper. Initial results were reported in Tenopir et al. [28], but analysis of the optional section was not included.

The study was approved as an anonymous online survey by the University of Tennessee Human Subjects Institutional Review Board. Respondents were free to withdraw from the survey at any time, and were not required to answer any questions to progress through the survey. An informed consent statement preceded the survey. Results were aggregated and no identifying information was collected.

Participant recruitment relied on snowball and volunteer sampling. An email was distributed by DataONE team members to contacts including deans, department chairpersons and program managers at various science agencies, major universities and research institutions around the world. The email to the contacts contained a link to the survey, which recipients were asked to forward and distribute to faculty, lecturers, post-doctoral research associates, graduate students, undergraduate students or researchers within their institution. In addition, the survey was distributed via a variety of environmental science listservs and blogs. Because of the openness of this recruitment approach, it is not possible to determine the response rate to the survey.

The survey was open for responses from 17 October 2013 to 19 March 2014. After eliminating respondents who answered only one or fewer questions, there were a total 1,015 responses to the full survey, of whom 595 answered the optional section that is analyzed in this paper. A copy of the survey without the optional section can be found as an appendix to Tenopir et al. [28]. The questions in the optional section are included in Appendix I of this paper. The survey data were archived as an SPSS file at Dryad after the initial publication [52] and were retrieved from the archive for analysis.

### Analysis approach

The data analysis proceeded in two stages. In stage one we developed scales for the different constructs of our theory through exploratory factor analysis and conceptual analysis of selected items of the survey. In stage two, we tested the relationships between the constructs using linear regression.

In the remainder of this section, we explain how we selected items from the survey to create scales to operationalize the constructs of the TRA model. We consider first the dependent variable, data reuse behaviour and then independent variables—attitudes and subjective norms—as well as control variables that might affect reuse.

#### Dependent variable: Data reuse behaviour

Several of the questions on the survey addressed data reuse behaviour. Question 47 asked about different approaches to obtaining data, asking “when I need to analyze data to answer a research questions, I…” with seven sub-questions about how respondents obtain their research data. We factor analyzed these items, choosing the number of factor to extract by examining the screeplot and applying a varimax orthogonal rotation. The sub-questions were distributed across three factors (Table 1), which we interpreted as collecting the data oneself (Factor 1), reusing already-collected data (i.e., data reuse, the behaviour of interest: Factor 2), and consulting an expert to obtain data (Factor 3).

**Table 1:** Factor loading for reported modes of obtaining data.

Variable	Factor 1	Factor 2	Factor 3
*Data collection Factor 1*: *Collect data oneself*			
Q47(1): make a plan to generate or collect the data I need myself.	0.4291		
Q47(2): make a plan to generate or collect the data I need within my research team.	0.4660		
*Data collection Factor 2*: *Reuse data*			
Q47(3): ask colleagues if they have data I can use for analysis.		0.7743	
Q47(4): search for data to use for analysis		0.6810	
Q47(5): ask colleagues if they know of data I can use for analysis		0.8519	
*Data collection Factor 3*: *Consult a professional*			
Q47(6): talk to a librarian about my data needs			0.5719
Q47(7): consult my data manager			0.5659

Orthogonal varimax rotation. Loadings < 0.3 not shown.

Question 48 asked for the frequency of the respondent ‘conducting research in which some or all of the data analyzed was collected by someone besides the respondent or members of the respondents’ immediate research team’ (measured on a Likert scale, 1 = never, 2 = seldom, 3 = sometimes, 4 = often, 5 = always).

When added to the factor analysis of Question 47, Question 48 (self-reported data reuse) aligned with Factor 2 in Table 1. This result suggested the creation of a composite scale for a construct for self-reported data reuse behaviour by averaging the responses to these four questions (Questions 47(3), 47(4) and 47(5) and Question 48). The averaging process has the advantage that it was robust to small amounts of missing data, e.g., if there was a missing response to one of the questions in a scale, we could use the average of the other questions to fill this gap. In other words, for this and all other scales, we handled missing data by replacing the missing item with the average of the other items in the scale. The alpha for this scale was 0.81, indicating that the scale for data reuse had good reliability.

#### Attitudes and subjective norms

The independent variables for this study were derived from survey questions that probed attitudes towards and subjective norms about data reuse. These included questions about attitudes towards conducting research in which some or all of the data were collected by others besides the researcher or members of the research team (Question 49), concerns that keep the respondent from conducting research in which some or all of the data analyzed were collected by others (Question 50) and as well two questions from the main survey that included sub-questions about attitudes towards the reuse of data (Question 20).

The relationships among the sub-questions were explored using factor analysis (following the process noted above) to get an initial sense of the structure of the data and the relationships among items. However, the primary approach to building scales was based on consideration of the theoretical constructs of TRA. We examined the meaning of the questions to identify what construct the sub-questions could be seen as measuring. Based on these two approaches, the first led by the data and the second driven by theory, we grouped sub-questions to form three scales for attitudes and two for subjective norms. A few sub-questions did not seem to fit either construct and so were not included in the analysis. The quality of the resulting scales was assessed by computing their reliability.

#### Data reuse attitudes

Several of the sub-questions reflected a perception that data reuse was easier or more efficient than other approaches (that is, it required less effort than the alternative). We created a scale by averaging together the selected sub-questions to form a scale, named Reuse Attitudes Factor 1 (abbreviated Reuse_ A_F1). The alpha for this scale was 0.79. The specific sub-questions included were:

- Q49(1): Data reuse saves time
- Q49(2): Data reuse is efficient
- Q49(3): Data reuse is easier than having to collect all my own data for analysis
- Q49(8): Data reuse is harder than conducting research using only my own data (reversed)
- Q49(9): Data reuse takes longer than conducting research with only my own data (reversed)

Second, two sub-questions reflected a perception of data reuse as being an effective way of conducting research, that is, that it led to better outcomes (abbreviated Reuse_A_F2). The alpha for this scale was 0.81. The specific sub-questions included were:

- Q49(6): Data reuse improves my results
- Q49(7): Data reuse helps me answer my research questions

Third, several sub-questions reflected concern about the trustworthiness of data or a lack of information about it (abbreviated Reuse_A_F3). The alpha for this scale was 0.73. The specific sub-questions included were:

- Q49(5): Data reuse requires too much trust in others’ methods
- Q50(2): I don’t trust others’ data collection methods
- Q50(3): Lack of adequate metadata
- Q50(5): I don’t have enough information about their data to feel confident using it

#### Data reuse norms

We next identified sub-questions that seemed to reflect subjective norms related to data reuse. The remaining sub-questions from Questions 49 and 50 seemed mostly to reflect negative perceived norms that might mitigate against data reuse, e.g. expressions that data reuse is not a known and accepted approach to conducting research. We used these sub-questions to form a scale for Reuse Norms, Factor 4 (abbreviated Reuse_N_F4), following the same procedure as above. The alpha for this scale was 0.74. The specific sub-questions included were:

- Q49(4): Data reuse is hard to explain in methods section
- Q50(1): I only receive career advancement credit for working with data I collect myself
- Q50(4): Not knowing how to share credit
- Q50(6): It’s too hard to explain in my research outputs
- Q50(8): I feel pressure to collect my own data

Finally, two of the sub-questions of Question 20 seemed to reflect perceptions of the importance of data reuse to advance science or a personal career, which we interpreted again as perceived importance of data reuse (Reuse_N_F5). The alpha for this scale was 0.80. The specific sub-questions included were:

- Q20(1): Lack of access to data generated by other researchers or institutions is a major impediment to progress in science.
- Q20(2): Lack of access to data generated by other researchers or institutions has restricted my ability to answer scientific questions.

#### Control variables

In addition to the main variables of the study, we included two sets of control variables in our analysis. First, previous studies have argued that some types of data are more likely or possible to be reused than others [53–56]. Some types of data are more sensitive and difficult to deal with, and thus, less likely to be shared and reused due to the practical and intellectual challenges involved [53–58]. Conversely, machine-generated data, such as hydrological, oceanic, atmospheric (upper and lower), and satellite data are easier to share and reuse than personally collected field or experimental data.

To account for such differences, as a control, we used responses from the main survey about the kinds of data used (Question 7, nine yes/no questions about the use of different kinds of data). To reduce the number of variables included in the analysis, the survey items about data were subjected to factor analysis, following the same process described above. This analysis suggested three factors (Table 2). These factors were interpreted as three main data types used by respondents: (1) models and remotely sensed data; (2) natural science surveys or observations; and (3) social science data. We then used regression to extract the three factors (abbreviated Data_F1 to Data_F3), and used those factors in subsequent analysis. Each factor is a normalized variable with a negative value for respondents who report little use of the related data type and a positive value for those who report much use.

**Table 2:** Factor loadings for types of data used.

Variable	Factor 1	Factor 2	Factor 3
*Data factor 1*: *Models and remote sensed data*			
Q7(3): Data models	0.3279		
Q7(7): Remote-sensed abiotic data (including meteorological data)	0.6915		
Q7(8): Remote-sensed biotic data	0.6448		
*Data factor 2*: *Natural science surveys or observation*			
Q7(1): Abiotic surveys (soils, microclimate, hydrology, etc.)		0.6011	
Q7(2): Biotic surveys		0.6300	
Q7(4): Experimental (involving some degree of manipulation)			
Q7(6): Observational (no manipulation involved)		0.3529	
*Data factor 3*: *Social science data*			
Q7(5): Interviews			0.6531
Q7(9): Social Science Survey			0.6247

Orthogonal varimax rotation. Loadings < 0.3 not shown.

Second, from the literature review, we considered that experience with use of data might be related to data reuse. As a proxy for experience with using data, we used as a measure, the self-reported use of metadata (Question 8), taking such use as an indication that the respondent had more developed data management practices.

Finally, as a comparison to data reuse, we examined self-reported data sharing behaviour. Data sharing was assessed from results found in the main part of the survey [28]. Two questions were asked directly about data sharing behaviour: Question 13, “How much of your data do you make available to others?” (1 = none, 2 = some, 3 = most or 4 = all); and Question 15(1), “I share my data with others” (1 = disagree strongly to 5 = agree strongly). The response to Question 13 was recoded to a 5-point scale (None = disagree strongly, some = disagree, most = agree or all = agree strongly), and then averaged with the response to Question 15(1) to create a scale for data sharing behaviour.

### Research model and hypotheses

**Fig 2:**
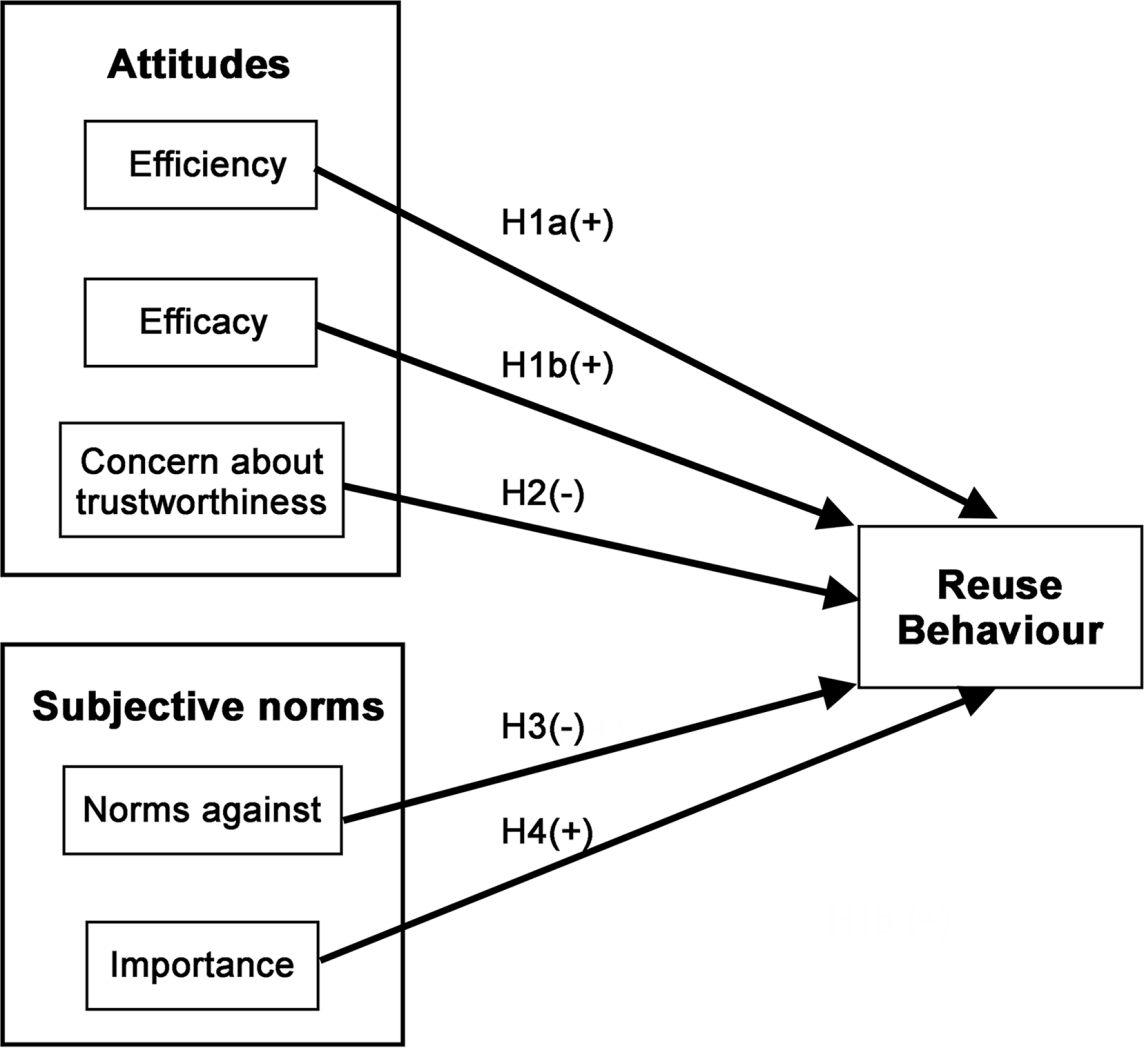
Research model. H1a: Perceived efficiency of data reuse will positively correlate with data reuse; H1b: Perceived efficacy of data reuse will positively correlate with data reuse; H2: Concerns about the trustworthiness of data will negatively correlate with data reuse; H3: Perceived norms against data reuse will negatively correlate with data reuse; and H4: Perceived importance of data reuse will positively correlate with data reuse.

The data analysis allowed us to map different sources of attitudes and subjective norms towards data reuse (Table 3), and to connect them to the underlying theory (Fig 1), to develop our research model (Fig 2).

**Table 3:** Relationship between TRA and research constructs.

TRA	Research Constructs	Factors	Survey Items
Attitudes Toward the Behaviour	Positive Attitudes Toward Data Reuse	Reuse_A_F1 Perceived efficiency of data reuse (positive effect)	Q49(1): Data reuse saves time
			Q49(2): Data reuse is efficient
			Q49(3): Data reuse is easier than having to collect all my own data for analysis
			Q49(8): Data reuse is harder than conducting research using only my own data (reversed)
			Q49(9): Data reuse takes longer than conducting research with only my own data (reversed)
		Reuse_A_F2 Perceived efficacy of data reuse (positive effect)	Q49(6): Data reuse improves my results
			Q49(7): Data reuse helps me answer my research questions
	Negative Attitudes Toward Data Reuse	Reuse_A_F3 Concern about trustworthiness of data (negative effect)	Q49(5): Data reuse requires too much trust in others’ methods
			Q50(2): I don’t trust others’ data collection methods
			Q50(3): Lack of adequate metadata
			Q50(5): I don’t have enough information about their data to feel confident using it
Subjective Norms	Subjective Norms	Reuse_N_F4 Perceived norms against data reuse (negative effect)	Q49(4): Data reuse is hard to explain in methods section
			Q50(1): I only receive career advancement credit for working with data I collect myself
			Q50(4): Not knowing how to share credit
			Q50(6): It’s too hard to explain in my research outputs
			Q50(8): I feel pressure to collect my own data
		Reuse_N_F5 Perceived importance of data reuse (positive effect)	Q20(1): Lack of access to data generated by other researchers or institutions is a major impediment to progress in science.
			Q20(2): Lack of access to data generated by other researchers or institutions has restricted my ability to answer scientific questions.
Intention	Not Measured	Not Measured	Not Measured
Behaviour	Reuse Behaviour	Reuse Self-reported reuse behaviour	Q47(3): ask colleagues if they have data I can use for analysis.
			Q47(4): search for data to use for analysis
			Q47(5): ask colleagues if they know of data I can use for analysis
			Q48: How often do you conduct research in which some or all of the data analyzed was collected by someone besides yourself or members of your immediate research team?

It is hypothesised that positive attitudes to data reuse are composed of beliefs about the perceived benefits due to (a) improved efficiency (Reuse_A_F1: the ability that data reuse will be performed by scientists with the least waste of time and effort), and (b) the efficacy of that reuse (Reuse_A_F2: the capacity of data reuse for producing the expected or desired results in research). We therefore hypothesize that:

H1: Perceptions that data reuse has benefits will positively correlate with data reuse.
H1a: Perceived efficiency of data reuse will positively correlate with data reuse.
H1b: Perceived efficacy of data reuse will positively correlate with data reuse.

It is hypothesized that negative attitudes are composed of the perceived risks associated with data reuse, in particular about the level of trustworthiness of the data (Reuse_A_F3: the perceived lack of credibility and reliability of the data). We therefore hypothesize that:

H2: Concerns about the trustworthiness of data will negatively correlate with data reuse.

It is hypothesized that negative attitudes to data reuse are composed of the subjective norms against data reuse (Reuse_N_F4: Perceived norms against data reuse), while positive attitudes are composed of the perceived importance of data reuse in the relevant community (Reuse N_F5: Perceived importance of data reuse). We therefore hypothesize that:

H3: Perceived norms against data reuse will negatively correlate with data reuse.
H4: Perceived importance of data reuse will positively correlate with data reuse.

The source data did not have usable measurements of intentions, so our research model was confined to testing the relationship between these measures of attitudes and subjective norms and the dependent variable (reuse behaviour).

## Results

A description of the sample and the descriptive statistics for the developed scales are provided first. The results of the regressions follow, first as they tested the research proposition, namely that attitudes and beliefs about data reuse are predictive of data reuse behaviour and second, to further explore the effects of developed data management practices.

### Descriptive statistics about respondents

Only the subset of people who answered the optional questions about data reuse are used here, so the reported distributions may differ from those reported by Tenopir et al. [28].

- Fifty-four percent of respondents came from the United States of America and the remaining 46% from across the globe (including Europe, China, Australia, South Africa and South America).
- The clear majority of respondents were academics (75%) but a substantial component were government employees (16%), and some commercial and non-profit agencies were represented.
- A range of disciplines were represented, from the natural sciences (50%) with the physical sciences (20%), health, social and humanities (12%), computing and information sciences (9%) education, law and business.
- Approximately 56% of respondents were researchers and analysts or senior academics, 17% early career academics (postdocs, lecturers or assistant professors) and 13% students (undergraduate and postgraduate). Only 2% of respondents were librarians or data managers, and 3% were retired. The remaining components were consultants or contractors (1%) and administrators or project managers making up 4%.

In summary, the sample was weighted towards respondents from the United States of America, in the natural sciences and at more senior positions, but includes a broad range of researchers.

### Descriptive statistics about behaviour, attitudes and subjective norms

#### Data reuse behaviour

**Fig 3:**
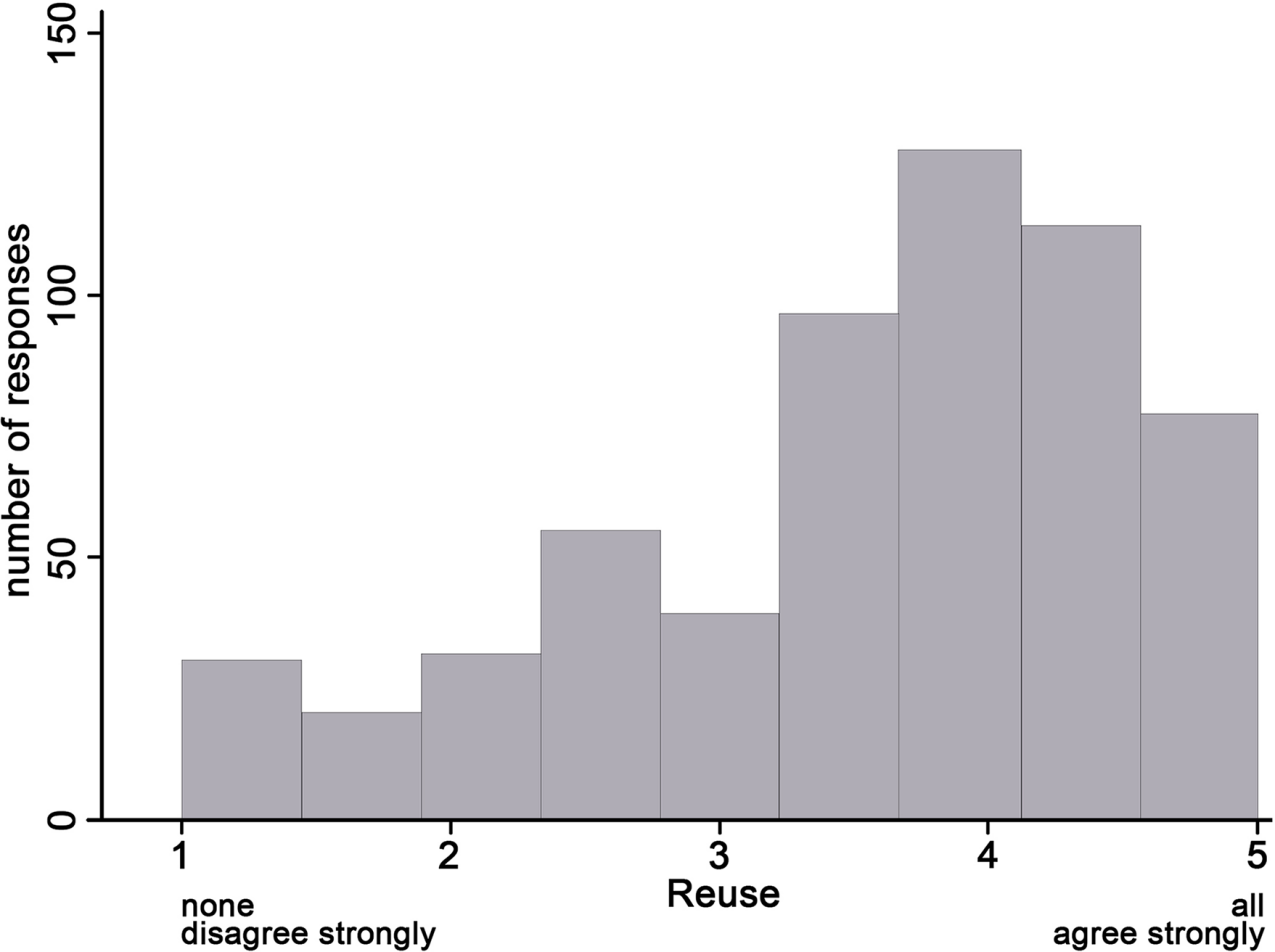
Distribution of self-reported data reuse behaviour. N = 589.

The dependent variable for our study was self-reported data reuse, an average of four survey items, on a scale from 1 (none/disagree strongly) to 5 (all/agree strongly). The mean score for the scale was 3.5 with a standard deviation of 1. The distribution was somewhat skewed to the positive (Fig 3). It may be that those who filled out the optional portion of the survey felt motivated to do so because they represent the fraction of survey respondents who engage more with reuse of data. Nevertheless, there was a distribution of self-reported reuse behaviour across the scale from none to all.

**Fig 4:**
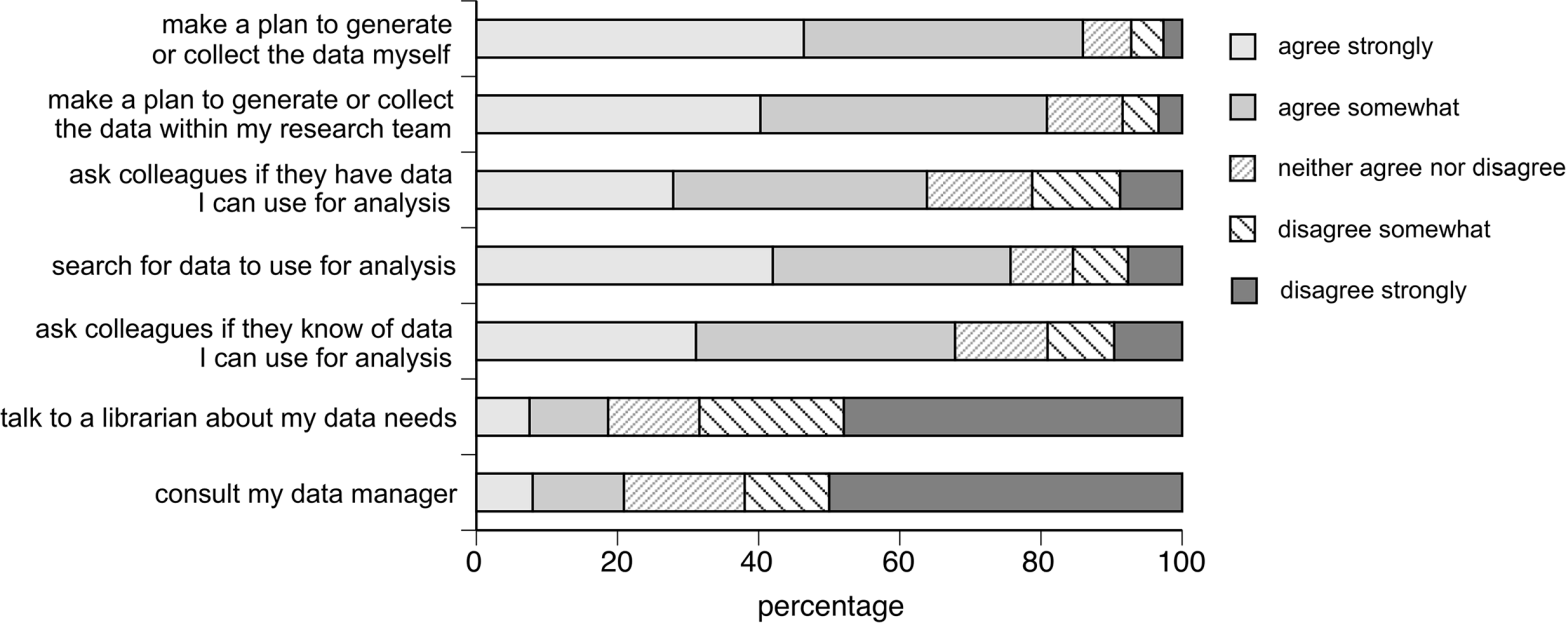
When I need to analyze data to answer a research question, I …(N = 569/ 569/ 570/ 576/ 572/ 557/ 526).

Responses to Question 47 split along two main lines. As shown in Fig 4, respondents strongly favoured collecting data themselves, from their team or from close colleagues, while equally strongly they did not consider it appropriate to ask a librarian or a data manager for (suitable) data. The only question in this suite suggesting data mining via the Internet (“I would search for data to use for analysis”) got an ambivalent response.

#### Data sharing behaviour

Respondents were asked to report how much data they shared on a scale of none, some, most, all. A few respondents said they did not share any data (10%). Most the respondents reported sharing some (43%) or most (32%) of their data with others, while 16% said they shared all their data (the percentages do not sum to 100% because of rounding).

Interestingly, the measures of self-reported data reuse and sharing behaviour were only moderately correlated, with a Pearson’s correlation of 0.25 (*p = 0*.*000)*, meaning that scientists who reportedly share their data are not necessarily data reusers and vice-versa.

#### Attitudes and subjective norms

The descriptive statistics for the variables in the study are shown in Table 4. Note that different scales have different numbers of missing data, as reflected in the different N for each variable. The statistics in the table indicate that most of the variables are slightly right skewed, but not so much as to threaten the planned analyses.

**Table 4:** Descriptive statistics for variables.

Variable	N	Mean	SD	Min	Max
Reuse—Self-reported reuse behaviour	589	3.527	0.995	1	5
Sharing—Self-reported sharing behaviour	592	3.538	1.098	1	5
Reuse_A_F1—Perceived efficiency of data reuse	580	3.238	0.840	1	5
Reuse_A_F2—Perceived efficacy of data reuse	572	3.568	0.827	1	5
Reuse_A_F3—Concern about trustworthiness of data	562	3.753	0.887	1	5
Reuse_N_F4—Perceived norms against data reuse	579	2.492	0.867	1	5
Reuse_N_F5—Perceived importance of data reuse	590	3.878	0.869	1	5
UseMetadata—Reported use of metadata	595	0.474	0.500	0	1
Data_F1—Use of models and remote sensed data	582	0.038	0.793	–0.813	1.692
Data_F2—Use of natural science surveys or observations	582	0.074	0.742	–0.901	1.492
Data_F3—Use of social science data	582	0.029	0.759	–0.636	1.840

### Predicting data reuse

The TRA hypothesizes that attitudes affect intentions and that intentions are predictors of behaviour. We lack explicit data on intentions, however, leaving us to test a direct relationship between attitudes and behaviour as shown in Fig 2. We tested this relationship using two linear regressions. First, we ran a regression including just the attitudes and subjective norms regarding data reuse to predict data reuse behaviour (Model 1 in Table 5). The R^2^ for this regression was 28%. The regression identified four data reuse factors as significant predictors of reuse behaviour (stars indicate coefficients significantly different from zero).

**Table 5:** Regression results.

	Model 1	Model 2
N	555	546
R^2^	0.28	0.36
Reuse_A_F1—Perceived efficiency of data reuse	0.111*	0.137**
Reuse_A_F2—Perceived efficacy of data reuse	0.260***	0.233***
Reuse_A_F3—Concern about trustworthiness of data	–0.015	–0.009
Reuse_N_F4—Perceived norms against data reuse	–0.112*	–0.097*
Reuse_N_F5—Perceived importance of data reuse	0.360***	0.299***
UseMetadata—Reported use of metadata		0.191**
Data_F1—Use of models and remote sensed data		0.283***
Data_F2—Use of natural science surveys or observations		–0.031
Data_F3—Use of social science data		–0.063

*** p ≤ 0.001.

** p ≤ 0.01.

* p ≤ 0.5.

+ p ≤ 0.1.

First, belief in the efficiency (Reuse_A_F1) and efficacy (Reuse_A_F2) of data reuse predicts data reuse, so H1a and H1b were supported. As well, perceptions of norms against data reuse (Reuse_N_F4) predicts less reuse (H2 was supported), while the perception of the importance of data reuse (Reuse_N_F5) predicts increased reuse (H4 was supported). Interestingly, agreement with concerns about the trustworthiness of data (Reuse_A_F3) did not predict less reuse of data, meaning that H3 was not supported. In summary, the data supported all but one hypothesis.

We then added the data and data management practice variables (Model 2 in Table 5). This regression had an R^2^ of 36%. For both regressions, we handled missing data using casewise deletion, resulting in a different N for the two. The four factors related to data reuse from Model 1 continued to be significant. In addition, use of remotely sensed data or data models was strongly associated with increased reuse, as was use of metadata.

### Differences between scientists according to data management experience

In the regressions reported above, we found that reported use of metadata was predictive of reuse. In the literature review, we noted the possibility that the importance of attitudes might also change with development of expertise using data (not just the value of the attitudes themselves). To examine this possibility, we divided the sample roughly into two based on reported use of metadata standards to describe one’s own data, used as a proxy for data experience. Again, missing data were handled by casewise deletion. The results of these regressions are shown in Table 6.

**Table 6:** Regression results comparing those who do or do not report using metadata.

	Don't use metadata	Do use metadata
	278	268
R^2^	0.37	0.26
Reuse_A_F1—Perceived efficiency of data reuse	0.074	0.198**
Reuse_A_F2—Perceived efficacy of data reuse	0.246***	0.217***
Reuse_A_F3—Concern about trustworthiness of data	–0.002	0.007
Reuse_N_F4—Perceived norms against data reuse	–0.132+	–0.070
Reuse_N_F5—Perceived importance of data reuse	0.368***	0.208***
Data_F1—Use of models and remote sensed data	0.301***	0.268***
Data_F2—Use of natural science surveys or observations	0.015	–0.058
Data_F3—Use of social science data	–0.057	–0.051

*** p ≤ 0.001.

** p ≤ 0.01.

* p ≤ 0.5.

+ p ≤ 0.1.

There is variation in the importance of factors between those who do and do not use metadata (e.g. the pattern in which factors are statistically significantly different than zero). We tested the statistical significance of the differences between the coefficients for the two groups by adding to the regression an interaction term between UseMetadata and the other factors. According to this test, only for Reuse_N_F5 are the coefficients significantly different between the two groups (a difference of 0.160, significant at p<0.10). The other differences between the coefficients for the two groups are smaller and so are not statistically significant.

## Discussion

The first observation from this analysis is that data reuse and data sharing are not linked, being only moderately correlated in our sample (r = 0.25). We interpret this as suggesting that the drivers of these two behaviours—attitudes, subjective norms and the kind of science that creates and that reuses data—are mostly distinct, leading to respondents displaying different behaviours for data sharing and reuse [51]. Although data sharing and reuse have important overlaps, they are not necessarily bounded by a cause-effect relationship. Some researchers may be data-sharers, but not re-users, or the other way around.

Second, our work confirms the proposition that attitudes towards and subjective norms about data reuse predict data reuse behaviour. The perceived efficacy of data reuse for answering research questions was found to be one of the strongest predictors of reuse behaviour. The perceived efficiency of data reuse had a positive effect on reported data reuse, but the effect is smaller than for perceived efficacy and importance of data reuse (Table 5).

When the sample was split according to use of metadata, perceived efficiency of data reuse remained positive, but was only significant for those who use metadata (Table 6). In other words, for researchers who are inexperienced in data management practices (as indicated by their non-use of metadata), perceptions of the efficiency of reuse seem not to be connected to actual behaviour. It could be that those with data management experience are forming their impressions of efficiency from experience, while those without form them *a priori*.

A common theme in the literature on data reuse has been the difficulty of being able to trust or even understand data produced by others. In contrast, the most striking result of our study is that expressed lack of trust in reused data was not a factor explaining a lack of data reuse. Indeed, many respondents agreed with questions about the lack of trustworthiness of data and still reported reusing data. This effect does not change with development of data management practices. It is difficult to unpack this result with the available data. It appears, however, that while respondents are aware of the potential pitfalls of reusing data, they apparently feel that they can overcome them in their own practice.

Turning to perceived norms, we found a large positive effect for the perceived importance of being able to reuse data. The effect was larger for those without developed data management practices (as indicated by knowledge and use of metadata). In contrast, there was a much weaker effect of perceived negative norms, the perception that data reuse is not accepted in the respondent’s field. Indeed, we found many respondents who agreed with statements such as “I only receive career advancement credit for working with data I collect myself” while still reporting high levels of data reuse.

As well, the effect of these perceived norms nearly disappears for those with developed data management practices, while the coefficient is negative and significant though only at p<0.10 for those who do not use metadata. As with the non-effect of trust, it appears that respondents (especially those with experience) are aware of the lack of acceptance of data reuse, but feel that they can overcome them. For example, scientists could be using pre-existing data with their own original data, thus obtaining the benefits of both approaches.

In addition to differences in perceived norms, fields differ in the kind of data used and the ease with which this data can be reused. In this regard, we found that reported use of models and remote-sensed data had a large positive effect on reuse behaviour. In the case of modelers, they require access to data to use in their models and do not generate their own data [59]. Remotely sensed data are collected via automated equipment, such as satellites, in contrast to data collected to individual scientists. These data are highly standardized and are captured and managed with the use of robust information technology [60]. These data are associated with little embedded intellectual property, are non-sensitive, and have a uniformity of format that allows them to be more easily reused that other types of data listed in the survey.

Finally, we note that the R^2^ for our regressions lie between 26–38%. While we explain some of the variation in reuse, more research is needed to identify the additional factors that drive scientists’ decisions about reusing data. Due to limitations in the available data, we connected attitudes directly to beliefs, rather than through an intermediate construct, ‘intention’, as the original TRA postulates. It could be that capturing the gap between intention and actual behaviour could provide a better fitting model.

### Challenges and limitations in reusing data

At this point, we reflect on the challenges of data reuse based on our own personal experiences in reusing data for this study. A first observation is that our experience matches reports in the literature about the difficulty of fitting already-collected data to new research questions. The survey we drew on was not originally designed to test our theoretical framework, leading to the need to reorganize the items to fit the model. Some items were written in a way that could not be easily plugged into our model. There is also a clear unbalance in the coverage of constructs that we are not able to fix by only reorganizing the items. For instance, while we have multiple items measuring some constructs, in other cases we have only a couple. And we lack data on intentions, a key construct in the TRA.

A further complication is that for the analysis we have created an additional layer of abstraction (construct–sub-category–item). For example, within attitudes, we have postulated a construct about attitudes about efficiency to which sub-questions are assigned. In other words, we have several scales that express different aspects of a given construct, but without those scales having been carefully planned up front for systematic coverage of the concept. Developing survey questions that systematically cover the different aspects of data reuse attitudes and subjective norms is another opportunity to improve the model.

Our experience analyzing the data also matches the survey questions about concerns with understanding and trusting the data. We faced challenges matching columns in the published dataset to questions on the survey and interpreting the numbers recorded (e.g. apparent inconsistencies in how responses to different questions were coded). Careful cross-checking of the results and the data stored in the file was needed to establish our confidence in our interpretation.

Despite these challenges, we also observe that this study achieved the expected benefits of data reuse. It was certainly more efficient to reuse the data from the existing survey that it would have been to run a new large-scale survey. And even with the limitations presented, the data were effective to answer the posed research questions, thus demonstrating the value of data reuse.

## Conclusion

This paper presents both theoretical and practical contributions to the study of scientific data reuse. In terms of theory, we developed a research model for how scientists’ normative perceptions and attitudes influence their data reuse behaviours. Therefore, this study offers a theory that can inform future studies on factors that may encourage or hinder scientists from reusing data collected by others.

We can draw some practical implications from our data to suggest what might be done to encourage scientists to reuse the increasing volumes of data being shared. First, given the importance of the perceived efficacy and to a lesser extent efficiency of data reuse, we suggest that these values be widely demonstrated to make them more apparent to more researchers. For example, a project like DataONE can provide concrete demonstrations of how data reuse can enable researchers to answer their current or new questions effectively and efficiently. Such demonstrations might take several forms, such as YouTube video case studies of data reuse; Jupyter notebooks demonstrating the process of reusing data; exemplary data reuse papers; or a combination of the above. Such materials could help reduce the initial barrier to data reuse by demonstrating in a more practical and palpable way its value. Additional materials could provide more specific training in the elements of data reuse, such as data discovery or use of metadata to help understand a dataset and its provenance. Another topic that seems to need better attention is data citation. Building and maintaining policies, guidelines and services for appropriate attribution and formal citation of datasets are key to leverage data sharing and to legitimize the reuse of these research artifacts.

While the literature emphasizes trust as an important factor in data reuse (or in deterring data reuse in the case of a lack of trust), this factor was not found to be significant in our study. It may be that the efforts of data repositories to establish the trustworthiness of candidate data for reuse and to encourage good metadata for shared data is paying off in the attitudes of the sample of scientists who completed the survey.

Finally, our results suggest the need to address norms about data reuse that encourage or discourage this practice. However, it must be recognized that norms are by their nature hard to change. Possible avenues for influence include having visible and established members of a field, publicly and also privately (e.g. through reviews or tenure letters) advocating for the value and acceptance of data reuse as a worthy research practice. Acceptability could be further shaped by recognition of good data reuse, such as awards for exemplary data reuse papers or other compensation mechanisms for those who manage expanding scientific discovery through reuse. Actions addressing attitudes and subjective norms to increase data reuse will be key to fully realizing the potential of research data and legitimize the investments and policies in favor of data sharing.

One location where data sharing and reuse are currently standard practice is the Synthesis Center. Synthesis Centers fund teams of people to tackle complex ecosystem science questions exclusively using existing data, and therefore can serve as a model for the promotion of data sharing and reuse in other scientific fields This replaces the model of the traditional research workflow which pivots around the collection of new data, with one of team science, giving ‘new life to old and dark data’ [19, 22, 34, 61, 62]. This has resulted in high productivity in terms of research outputs; in fact, the Synthesis Centre NCEAS, is one of the most highly cited research units in the United States of America, with over 2,500 articles [63] across its more than 20 years of existence, 12% in high impact journals.

There is promising evidence both here and in previous research that the case for data reuse is being heard. Incentivizing and normalizing this emerging model of research practice remain fruitful areas of inquiry, as well as the need to bridge the apparent gap between sharing and reuse behaviours.

Since this research was based on published dataset a next step for advancing towards a more comprehensive understanding of the reuse of data would be to identify other dimensions and factors that may prevent and/or prompt scientists’ data reuse behaviours. This study encourages future research to consider not only reusing the theoretical model and measurements proposed by this study, but to expand on them, by adding constructs such as intention to reuse, and facets this research might have overlooked or that could not be assessed due to the limitations of the data at hand. Finally, future research should examine the antecedent of attitudes and subjective norms suggested by TRA to determine which are most influential, to develop recommendations that address these factors and so promote data reuse.

## Appendix I. Questions from optional survey section

**46)** To also help us serve scientists’ research process needs, would you be willing to answer 4 additional short questions?
  o Yes
  o No
          If No, go to Final Web Page
**47) Tell us how much you agree with the following ways to complete this sentence:When I need to analyze data to answer a research question, I ……**.

**Table:** 

	agree strongly	agree somewhat	neither agree nor disagree	disagree somewhat	disagree strongly	not sure
1) …….make a plan to generate or collect the data I need myself.	o	o	o	o	o	o
2) …….make a plan to generate or collect the data I need within my research team.	o	o	o	o	o	o
3) …….ask colleagues if they have data I can use for analysis.	o	o	o	o	o	o
4) …….search for data to use for analysis.	o	o	o	o	o	o
5) ……. ask colleagues if they know of data I can use for analysis.	o	o	o	o	o	o
6) …….talk to a librarian about my data needs.	o	o	o	o	o	o
7) …….consult my data manager.	o	o	o	o	o	o

48) How often do you conduct research in which **some or all of the data analyzed was collected by someone besides yourself or members of your immediate research team**? (Choose the one best answer.)
  o Never
  o Seldom
  o Occasionally
  o Frequently
  o Always
**49) Tell us how much you agree with the following ways to complete this sentence**:
**Conducting research in which some or all of the data analyzed was collected by others besides myself or members of my immediate research team……**.

**Table:** 

	agree strongly	agree somewhat	neither agree nor disagree	disagree somewhat	disagree strongly	not sure
1) …….saves time.	o	o	o	o	o	o
2) …….is efficient.	o	o	o	o	o	o
3) …….is easier than having to collect all my own data for analysis.	o	o	o	o	o	o
4) …….is hard to explain in methods section.	o	o	o	o	o	o
5) …….requires too much trust in others’ methods.	o	o	o	o	o	o
6) …….improves my results.	o	o	o	o	o	o
7) …….helps me answer my research questions.	o	o	o	o	o	o
8) …….is harder than conducting research using only my own data.	o	o	o	o	o	o
9) …….takes longer than conducting research with only my own data.	o	o	o	o	o	o

**50) Tell us what concerns keep you from conducting research in which some or all of the data analyzed was collected by others besides yourself or members of your immediate research team**.

**Table:** 

	agree strongly	agree somewhat	neither agree nor disagree	disagree somewhat	disagree strongly	not sure
1) I only receive career advancement credit for working with data I collect myself.	o	o	o	o	o	o
2) I don’t trust others’ data collection methods.	o	o	o	o	o	o
3) Lack of adequate metadata.	o	o	o	o	o	o
4) Not knowing how to share credit.	o	o	o	o	o	o
5) I don’t have enough information about their data to feel confident using it.	o	o	o	o	o	o
6) It’s too hard to explain in my research outputs.	o	o	o	o	o	o
7) I don’t like working with others.	o	o	o	o	o	o
8) I feel pressure to collect my own data.	o	o	o	o	o	o
9) Other (please specify below).	o	o	o	o	o	o

If you selected other, please specify:
